# Measuring and correcting bias in indirect estimates of under-5 mortality in populations affected by HIV/AIDS: a simulation study

**DOI:** 10.1186/s12889-019-7780-3

**Published:** 2019-11-12

**Authors:** John Quattrochi, Joshua A. Salomon, Kenneth Hill, Marcia C. Castro

**Affiliations:** 1 Department of Public Health, Simmons University, 300 The Fenway, Boston, MA 02115 USA; 2 Center for Health Policy and Center for Primary Care Outcomes and Research, Stanford University, 616 Serra Street, Stanford, CA 94305 USA; 3 Department of Global Health and Population, Harvard T.H. Chan School of Public Health, 655 Huntington Ave., Boston, MA 02115 USA

**Keywords:** Under-5 mortality, Indirect methods of estimation, Bias, HIV/AIDS

## Abstract

**Background:**

In populations that lack vital registration systems, under-5 mortality (U5M) is commonly estimated using survey-based approaches, including indirect methods. One assumption of indirect methods is that a mother’s survival and her children’s survival are not correlated, but in populations affected by HIV/AIDS this assumption is violated, and thus indirect estimates are biased. Our goal was to estimate the magnitude of the bias, and to create a predictive model to correct it.

**Methods:**

We used an individual-level, discrete time-step simulation model to measure how the bias in indirect estimates of U5M changes under various fertility rates, mortality rates, HIV/AIDS rates, and levels of antiretroviral therapy. We simulated 4480 populations in total and measured the amount of bias in U5M due to HIV/AIDS. We also developed a generalized linear model via penalized maximum likelihood to correct this bias.

**Results:**

We found that indirect methods can underestimate U5M by 0–41% in populations with HIV prevalence of 0–40%. Applying our model to 2010 survey data from Malawi and Tanzania, we show that indirect methods would underestimate U5M by up to 7.7% in those countries at that time. Our best fitting model to correct bias in U5M had a root median square error of 0.0012.

**Conclusions:**

Indirect estimates of U5M can be significantly biased in populations affected by HIV/AIDS. Our predictive model allows scholars and practitioners to correct that bias using commonly measured population characteristics. Policies and programs based on indirect estimates of U5M in populations with generalized HIV epidemics may need to be reevaluated after accounting for estimation bias.

## Background

Under-5 mortality (U5M) is an important indicator of population health, and relationships between U5M and fertility, population growth, economic growth, and democratization are actively researched [1–6]. Several national and international goals, most notably the Millennium Development Goals (MDGs) and the Sustainable Development Goals (SDGs), have included U5M as a target indicator. MDG4 called for a 2/3 reduction from 1990 U5M levels by 2015, and SDG3 calls for a reduction of U5M to at least 25 per 1000 live births by 2030. Yet accurate measurement of U5M in many countries is still hampered by the quality and/or availability of data [7–10].

Most child deaths occur in countries that lack or have incomplete vital registration systems. In such populations, survey- and census-based methods for mortality rate estimation are commonly used. Survey-based methods include direct and indirect estimation. The former requires the collection of a full birth history, that is, date of birth and age at death, if appropriate, for every live birth a woman has had. With that information U5M rates can be calculated for any time period before the survey. However, because of small sample sizes, rates are typically calculated for 5-year periods (1–5, 6–10 and 11–15 years before the survey). Indirect methods, by contrast, require only the collection of a summary birth history [11]. Mothers are asked about the number of live-born children they have ever given birth to and the number that are still alive. No information about dates of birth or dates of death is collected. Models of fertility and age-specific mortality are used to estimate the probability of dying between birth and age 5 (U5M) based on the ratio of children dead (CD) to children ever born (CEB). The resulting estimates correspond to periods that precede the survey date by a length of time determined largely by age patterns of fertility, approximated by parity ratios across age groups [12]. Although full birth histories have come to dominate the measurement of U5M at the country level, summary birth histories remain valuable. They are often included in population censuses, and offer greater potential for spatial or socioeconomic disaggregation [13].

In populations affected by HIV/AIDS, three key assumptions of indirect methods for U5M estimation are likely to be violated. First, the methods assume that the survival of a mother and the survival of her children are not correlated. HIV/AIDS has a substantial impact on the mortality risks of children born to HIV positive mothers due to vertical transmission of the virus and to other harmful consequences of maternal death. Empirical studies demonstrate that the survival of a mother and that of her children are highly correlated in populations affected by HIV/AIDS [14]. Note that this also leads to bias in *direct* estimates of U5M that rely on surveys, because women who have died are under-represented in the survey sample.

The second assumption is that the mortality experience of the children of mothers in each age group at the time of the survey is representative of the mortality experience of the children of all mothers for some time period in the past; in other words, time trends in U5M need to have been gradual and unidirectional. If the incidence of HIV/AIDS has changed over time (or access to antiretroviral therapy (ART) has changed) then this assumption would be violated.

The third assumption is that age-patterns of under-5 mortality are accurately captured in the mortality model (i.e., life table) that is used. To the extent that populations impacted by HIV are likely to have age-patterns of mortality that differ from those available in any model life tables, then the indirect estimates would be biased.. Recently developed model life tables based on demographic surveillance systems in rural Africa are among the first to account for the impact of HIV [15].

Underestimation of U5M may have a range of undesirable consequences. First, it can lead to overestimates of intervention effectiveness and to false declarations of success in campaigns to meet objectives such as the MDGs or the SDGs. If the bias is large enough, it may appear that U5M is decreasing when it is in fact increasing. Second, it may also result in resources previously dedicated to lowering U5M being reallocated to other targets when there is still scope for these resources to produce significant benefits in reducing the burden of U5M. Finally, underestimates of U5M may make epidemics, such as HIV, appear less harmful than they are in reality. To address these concerns, we offer an alternative to correct the bias due to HIV in indirect estimates of U5M, which requires only estimates of HIV prevalence in the year of the survey and 10 years prior to the survey, and an estimate of ART prevalence in the year prior to the survey. Given the centrality of U5M estimates to many policy and planning efforts in global health, we intend that this tool will facilitate more reliable U5M estimation for countries impacted by HIV and produce corresponding benefits for priority-setting and other decision-making in these settings.

Previous studies of the bias in estimates of U5M due to HIV/AIDS include [16–18]. Only Ward and Zaba [16] assessed indirect estimates, using a stable population model, and assuming that HIV incidence was stable over time. They found that the degree of negative bias in indirect mortality estimates increased from 1.2 to 44.3% as the adult prevalence of HIV increased from 2.5 to 45%, with greater bias in estimates from older women, particularly those aged 45–49.

Hallett et al. [17] calculated bias in direct estimates of U5M based on a prospective, population-based cohort in rural Zimbabwe that used verbal autopsies to identify AIDS deaths. They also built a mathematical model calibrated to the empirical data to estimate and correct the bias in U5M. Bias was calculated by comparing a demographic and health survey (DHS) continuous time series, consisting of smoothed direct estimates of U5M, to a DHS corrected time series. Reports from surviving mothers underestimated U5M by 9.8% compared to reports from all mothers, in a population in which HIV prevalence fell from 22% in 1998 to 18% in 2005.

Most recently, Walker et al. [18] used a cohort component projection model where the key inputs were derived from the latest projections available from the Joint United Nations Programme on HIV/AIDS (UNAIDS) Spectrum package [19]. Spectrum outputs include: annual number of births (typically from 1970 onwards), number of women each year in need of prevention of mother-to-child transmission (PMTCT - considered as a proxy for the number of births to HIV-positive women), and number of HIV-positive infants. The Spectrum model takes into account the fertility-reducing effects of HIV, the estimated transmission of HIV from mother to child, breastfeeding patterns, and the impact of interventions to reduce MTCT. For HIV-negative births, the risks of dying in each year from birth to age 5 years were obtained from a model life table in the Coale and Demeny “West” family, using a level of U5M that was a best guess of the U5M in the HIV-negative population. Thus, the model assumed that mortality of HIV-negative children born to HIV-positive mothers was the same as that for children born to HIV-negative mothers. The model did not take into account the age when a woman is infected with HIV when estimating mortality due to AIDS. It estimated bias by comparing the ratio of under-five deaths to births for all mothers and for surviving mothers across the 35-year intervals preceding the year of the survey.

This paper builds on the literature examining bias in U5M estimates, focusing on indirect methods and using a simulation model to incorporate a more comprehensive set of population characteristics than in previous studies. Using the model to simulate a variety of trajectories in HIV incidence, levels of ART coverage, mortality rates and fertility rates, we calculated the magnitude of bias in indirect estimates of U5M under different combinations of these variables. Based on the results of the simulations, we developed a parsimonious predictive model of bias as a function of a subset of these variables, and we used the predictive model to adjust estimates based on empirical data from Malawi and Tanzania. This analysis was the first since Ward and Zaba [16] to assess indirect estimates. Unlike Ward and Zaba [16], the evolution of the AIDS epidemic was incorporated into the simulation model, and unlike Walker et al. [18] the dynamics of ART take-up were included. In addition, the simulation used more recent data than Ward and Zaba [16] and Hallett et al. [17], and, unlike the latter, it was not calibrated to empirical cohort data, which means that this study relies more on parameters estimated in previous studies.

## Methods

### Simulation methodology

We created a discrete-time, stochastic, individual-based model to simulate fertility, HIV infection, ART initiation, and mortality for women and their children living during the period 1946–2010. In each yearly time step, each woman in the model faces some probability of giving birth, being infected with HIV, initiating ART (if HIV-positive), and dying. Children born to HIV-positive mothers face some probability of infection at birth, all children face some probability of dying each year, and female children, should they survive to age 15, begin to face the same probabilities listed above. In other words, children born during the simulation can become adults in the simulation. Parameters of the model were derived from published and unpublished sources, as detailed below. Some of the parameters (the “inputs”) were varied across simulations in order to generate populations with a wide range of fertility, mortality, HIV incidence, and ART initiation trajectories. Other parameters remained fixed across populations, particularly those that define biological relationships (e.g. survival time among HIV-positive women who do not initiate ART).

The goal of the simulation was to create a wide variety of population histories, resembling the experiences of different actual populations, to assess how bias will vary in relation to other population characteristics that may be measured independently (e.g., HIV prevalence). In order to characterize these general relationships rather than their expression in a small number of particular populations, the parameters included in the simulation model vary over a range of different values that each selected population characteristics may take, rather than precisely matching fertility, mortality, HIV incidence, and ART initiation rates experienced in specific settings. All simulations were run in R [20], and the data and code are freely available at https://github.com/jquattro/hiv-childmort-bias. A user-friendly web application to correct indirect estimates is available at johnquattrochi.com/bias.

### Simulation parameters

#### Size and date of initial population

We initiated the simulation with 22,500 women who were aged 15 years in 1906, and ran the simulation through 2010. This was the smallest initial population and shortest simulation duration (104 years) that produced stable estimates. Larger initial populations and longer durations were too computationally costly.

#### Annual probability of birth, HIV negative women

We defined the annual probability of birth as a function of calendar year and mother’s age. The birth probability was set to zero for women younger than 15 years and older than 49 years. We used estimates of age-specific fertility rates (ASFR) from the United Nations Population Division’s World Fertility Data [21], which provided estimates for years when surveys or censuses are available (roughly every 5 years). For years when ASFR were not available, we adjusted the nearest available ASFR using the interpolated estimates of the total fertility rate (TFR) from the United Nations Population Division’s World Population Prospects [22]:

$$ \Pr {(birth)}_{current\ year, age, input}={ASFR}_{nearest\ year, input}\ast \left(\frac{TFR_{current\ year, input}}{TFR_{nearest\ year, input}}\right) $$

where: *current year* is the current year in the simulation; *nearest year* is the year nearest to the current year for which ASFR are available; *age* is age of mother in current year; and *input* is the country from which fertility data is being used for the current simulation. To account for postpartum amenorrhea, we divide the probability of birth by two in the year following a birth.

#### Annual probability of birth, HIV positive women not on ART

Using DHS data, Chen and Walker [23] found that among women aged 15–19 years, those who were HIV-positive experienced higher ASFRs compared to HIV-negative women, with the ratio dependent on the percent of 15–19 year old women who were sexually active; also, among those aged 19, HIV-positive women experienced lower fertility rates relative to HIV-negative women. We use the ratios estimated by Chen and Walker [23] as fixed parameters in the simulation model (although the percent of females aged 15–19 who are sexually active was an input that varied across simulations.

#### Annual probability of birth, HIV positive women on ART

Several studies have found that incidence of pregnancy increases following initiation of ART [24–26], while at least one has found that incidence does not increase [27]. The effect of ART on fertility likely depends on age, cluster of differentiation 4 (CD4) count at initiation, educational attainment, contraceptive use, and partner’s HIV status. For the simulation model, we assumed that, among women over age 19, ART erases half of the fertility decrease caused by HIV/AIDS. In other words, for women on ART, the ASFR ratios in Chen & Walker [23] increase by half the difference from one (one indicating equal ASFRs between HIV-positive and HIV-negative women). We assumed that the ASFR for 15–19 year olds is not affected by ART. This simplifying assumption has minimal effect as few women in the simulation will be infected with HIV/AIDS and initiate ART by age 19.

#### Maternal mortality: probability of mother’s death at each birth

Inputs relating to maternal mortality included the maternal mortality ratio (MMR - maternal deaths per 100,000 births) in 1990 and the annual decline in MMR since 1990. The initial value of the MMR was either 0.0012 or 0.012, representing the range of empirical estimates from Hogan et al. [28]. For similar reasons, the annual rate of decline was set to 0 or 7.3%. Blanc, Winfrey, and Ross [29], using data from 38 DHS, found that MMR had a J-shaped relationship with age; women aged 40–49 experienced an MMR roughly 3 times greater than women aged 20–24, while women aged 15–19 experienced an MMR roughly 20% greater than women aged 20–24. For the sake of model parsimony, we ignored the higher risk for younger women. For women aged 25 years and younger, the risk of death at each birth was equal to the MMR divided by 100,000. For women older than 25 years, the risk of death was assumed to be:

$$ \mathit{\Pr}\left( death| birth\right)=\frac{MMR_{input, year}}{\mathrm{100,000}}+\left(\frac{\left( age-25\right)}{25}\right)\ast 2\ast \frac{MMR_{input, year}}{\mathrm{100,000}}, $$

where: *input* is the input series of MMRs based on Hogan et al. [28]; and *year* is the current year in the simulation. The per-birth probability of maternal mortality in HIV-positive women was set at 8.2 times greater than the probability for HIV-negative women based on Zaba et al. [30].

#### Annual probability of HIV infection

The annual probability of HIV infection was selected among the HIV incidence curves estimated by Hogan and Salomon [31] for 31 African countries. We selected five curves that included early-starting and late-starting epidemics, with either high or low peak incidence. The age pattern of incidence was determined using age-specific HIV incidence ratios from Heuveline [32].

#### CD4 count at infection and annual progression of CD4 count

Parameters governing CD4 count were derived from Hallett et al. [33]. Specifically, when a woman was infected with HIV, the square root of her initial CD4 count was a random draw from a normal distribution with a mean of 25.9 and a standard deviation of 0.61. CD4 was assumed to decline linearly over time. For each woman under age 35 the absolute yearly decline was defined by a random draw from a normal distribution with a mean of 1.32, and a standard deviation of 1. For women 35 years or older the draw came from a normal distribution with a mean of 2.0 and a standard deviation of 1.

#### Annual probability of ART initiation, given that CD4 < threshold

We used World Development Indicator (WDI) data on ART coverage for 2009 and 2011 for selected countries [34]. We assumed that coverage was 0 in 2004 and we linearly interpolated coverage levels for 2005 to 2008, and again for 2010. In the WDI data, ART coverage is expressed as a prevalence measure, i.e. the ratio of the number of people receiving ART to the number of people eligible to receive ART. We converted prevalence to incidence using a simplifying approximation based on the equilibrium relationship:

$$ \mathrm{Prevalence}=\mathrm{incidence}\ast \mathrm{duration} $$

For duration, we assumed that the median survival time on ART is 13 years [33]. Thus we ended up with a series of annual probabilities for initiating ART given that a woman’s CD4 was below threshold, for 2004 to 2010.

#### Annual probability of death, HIV negative individuals

Time series for _5_q_0_ and _1_q_0_ estimates from the UN Inter-agency Group for Child Mortality Estimation (IGME) for selected countries were used as inputs [35]. To estimate one-year, age-specific probabilities of death, the ratios of _1_q_2_ to _1_q_3_ to _1_q_4_ from the UN Model Life Table, General Pattern for both sexes, were used to interpolate from the IGME estimates.

Time series for the probability of dying between ages 15 and 60 (_45_q_15_) were taken from the Institute for Health Metrics and Evaluation (2010) for selected countries. To obtain age-specific annual probabilities of death from ages five and up, the _45_q_15_ for an input “model country” and year in the simulation were matched to the UN model life table with the closest _45_q_15_ [22].

#### Annual probability of death, HIV positive individuals not on ART

The annual probability of death for HIV-positive women who were not on ART was based on cumulative mortality reported in Walker, Hill, and Zhao [18], who drew on cohort studies by Schneider, Zwahlen, and Egger [36], Todd et al. [37], and Stover et al. [38].

#### Annual probability of death, HIV positive women on ART

HIV-positive women on ART faced an annual probability of death that was a function of CD4 count at ART initiation, presence or absence of symptoms at baseline, and time since initiation. The function was taken from the “medium” scenario published by Hallett et al. [33]. Women were assigned to “symptomatic” or “non-symptomatic” with probability 0.5, based on Braitstein et al. [39]. The median survival after initiation of ART ranged from roughly 13 to 19 years.

#### Mother-to-child transmission of HIV

Probability of mother-to-child transmission of HIV was taken from Stover et al. [38] Transmission depends on breastfeeding duration and ART, including the assumption that all ART is single-dose nevirapine, which is less effective at preventing transmission than dual- or triple-treatment ART.

### Range of inputs used in the simulation

The primary goal was to measure bias in indirect estimates across a set of populations that have experienced different rates of fertility, mortality, HIV infection, and ART initiation. To generate such a set of populations, we varied ten inputs: fertility, adult mortality, U5M, percent of 15–19 year olds who are sexually active, maternal mortality in 1990, percent annual decline in the maternal mortality rate, HIV incidence, duration of breastfeeding, and ART coverage. We simulated one population for each combination of inputs, for a total of 4480 populations.

**Fig. 1:**
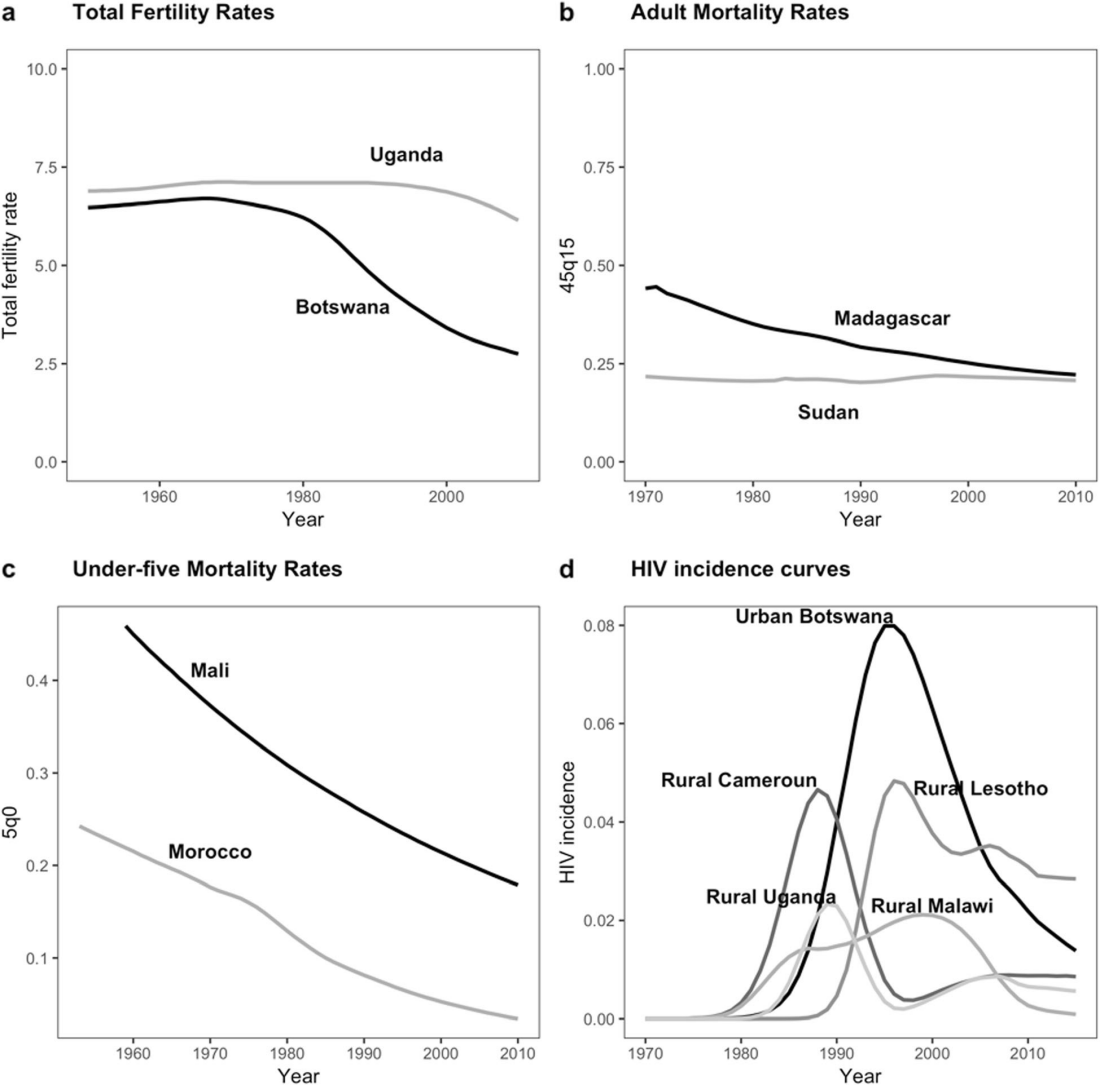
Inputs used in the simulation: total fertility rates, adult and under-five mortality rates, and HIV incidence rates from selected countries. Notes: **a** TFR estimates for Uganda and Botswana [21]; **b** 45q15 estimates for Madagascar and Sudan [40]; **c** U5M estimates for Mali and Morocco [35]; **d** estimates of HIV incidence for rural Cameroon, rural Lesotho, rural Malawi, rural Uganda, and urban Botswana [31]

With regards to fertility, we considered a time series of TFR estimated by the UN Population Division [21]. We selected Botswana and Uganda (Fig. 1a) in order to have populations with high but declining fertility or with stable high fertility, reflecting the experience of many developing countries.

For adult mortality we considered IHME estimates of _45_*q*_15_ for 195 countries, 1970–2010 [40]. We selected Madagascar and Sudan to represent high-and-decreasing and low-and-steady adult mortality (Fig. 1b).

For U5M we considered UN IGME [35] estimates for 195 countries. We chose estimates for Mali and Morocco to represent high-and-decreasing and low-and-decreasing U5M, in populations with low prevalence of HIV/AIDS (Fig. 1c). Note that, in the simulation, these are background mortality rates that capture causes of death other than HIV/AIDS.

For HIV incidence, we considered 31 curves estimated for urban or rural parts of selected African countries [31]. We chose curves for urban Botswana, rural Cameroon, rural Malawi, rural Lesotho, and rural Uganda to vary the timing of epidemic onset and the level of epidemic peak (Fig. 1d).

**Fig. 2:**
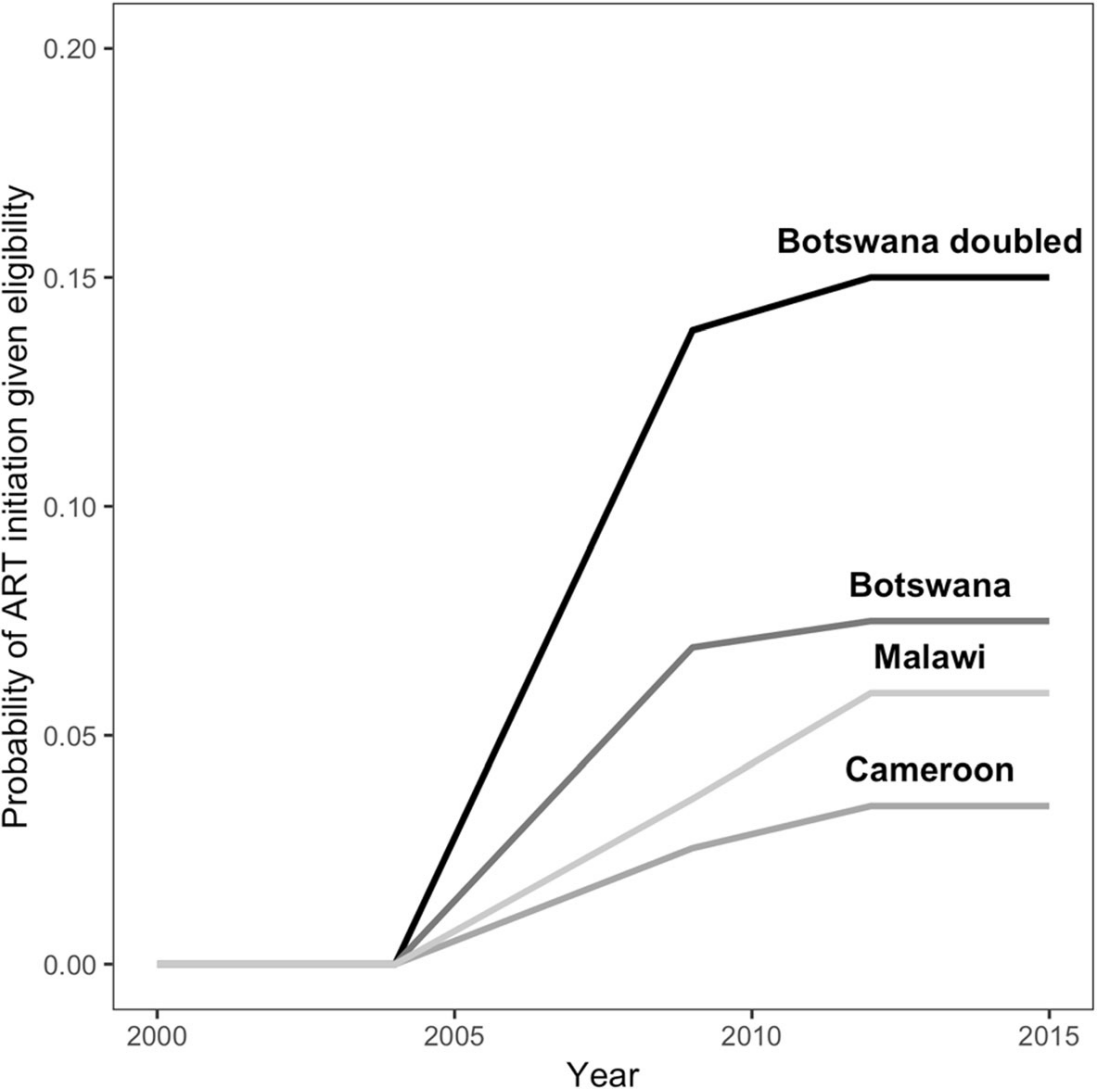
Probabilities of anti-retroviral therapy initiation used in simulations. Notes: Annual probability of initiating ART in simulation model if individual has a CD4 count below the treatment threshold, based on World Development Indicator [34] data on ART coverage for 2009 and 2011 for Botswana, Cameroon, and Malawi

National estimates of the rate of ART uptake given CD4 below a treatment threshold are not available. Therefore we used WDI [34] estimates of ART coverage for Botswana, Cameroon, and Malawi to calculate a reasonable set of probabilities of ART initiation (Fig. 2). We added the highest curve based on twice the ART coverage in Botswana to cover populations that experience particularly rapid uptake.

### Indirect estimation of under-5 mortality and calculation of bias

For each simulated population, we tabulated CEB and CS as of 2010 for two overlapping groups of women: (1) all surviving women aged 15–49, and (2) all surviving women and all women who died from HIV/AIDS aged 15–49. We used all women in each category rather than drawing a sample to simulate a survey in order to avoid sampling variability and focus on bias due to HIV/AIDS. The second population approximates a counterfactual in which no bias due to HIV/AIDS occurs. Inherent in our tabulations is the assumption that ‘dead’ women provide equally valid responses as women who survived. For each of the two groups of women, we used indirect methods to estimate under-5 mortality for each of the 75-year age groups of mothers aged 15–49 years [11]. We used a UN General Standard model life table to estimate _n_q_0_ and to convert _n_q_0_ into _5_q_0_.

We defined bias in two ways:

$$ Relative\ bias=\frac{IE_{survivors}-{IE}_{survivors\& HIV\  deaths}}{IE_{survivors\& HIV\  deaths}} $$

$$ Absolute\ bias={IE}_{survivors}-{IE}_{survivors\& HIV\  deaths} $$

where *IE*_*survivors*_ was indirect estimates of U5M using women who were alive in 2010, and *IE*_*survivors* & *HIV deaths*_ was indirect estimates of U5M using women who were alive in 2010 and women who died from HIV/AIDS prior to 2010 but would have been 15–49 in 2010 had they survived.

### Predictive model to correct for bias from HIV mortality

Our aim was to develop a predictive model, based on a large number of simulations, which related the bias due to HIV/AIDS in indirect measures of U5M to a small number of predictor variables that are available for most countries. The dependent variable was the absolute bias as defined above; the unit of analysis was the simulated population of a particular age group.

We employed a variety of modeling strategies, drawing on recent developments in predictive modeling [41]. We randomly selected 80% of our data for model fitting, and used the other 20% for out-of-sample predictions. We gauged model performance using four metrics of out-of-sample prediction accuracy: root mean squared error, root median squared error, mean relative error, and median relative error.

The full model included 53 variables: unadjusted U5M; five-year age group dummies; HIV prevalence 5, 10, and 20 years before the survey; ART prevalence 1, 3, and 5 years before the survey; TFR in the year of the survey and 10 years earlier; interactions between HIV prevalence and age group; interactions between ART prevalence and age group; and an intercept term. Note that while 2010 is used as the year of the survey throughout this paper, the predictive equation can be used for other years.

Our modeling strategies included forward and backward selection, principle components regression, partial least squares regression, and generalized linear models with penalized maximum likelihood. For forward and backward selection, we used Akaike’s Information Criterion and a Bayesian Information Criterion [42]. We fit principle components regressions with 20, 30, and 35 components, and we fit partial least squares regressions with 16 and 32 components. We also fit a generalized linear model via penalized maximum likelihood with three elastic-net penalties: 0 (commonly referred to as ridge regression), 1 (lasso), and 0.5 (an intermediate value). With the penalty at zero, the coefficients of correlated predictors shrink towards zero and each other. With the penalty at one, a single coefficient will be retained from a group of correlated predictors. We used 10-fold cross-validation to select the elastic-net tuning parameter, and we generated prediction intervals from the generalized linear models via bootstrapping.

### Application to empirical data from Malawi and Tanzania

We applied the best-performing model (lasso regression; see Table 4) to empirical data from Malawi and Tanzania to correct for bias in U5M. These countries were chosen because they include relatively high U5M and HIV prevalence (Table 1). Data were assembled from different sources: CEB, CD, and TFR came from the 2010 DHS [43, 44]; estimates of HIV prevalence came from UNAIDS [45]; number of women on ART and ART coverage came from WHO/UNICEF/UNAIDS [46] and national reports [47, 48]; and population totals came from World Population Prospects [22].

**Table 1:** Child survival, HIV, ART, and TFR for Malawi and Tanzania

		Malawi	Tanzania
	*Mother’s age*		
Children ever born	15–19	0.23	0.20
	20–24	1.61	1.38
	25–29	2.98	2.67
	30–34	4.23	3.66
	35–39	5.45	5.03
	40–44	6.26	5.66
	45–49	6.91	6.35
Children surviving	15–19	0.21	0.19
	20–24	1.44	1.28
	25–29	2.64	2.42
	30–34	3.59	3.27
	35–39	4.50	4.34
	40–44	5.04	4.87
	45–49	5.29	5.28
HIV prevalence, 1990		0.072	0.048
HIV prevalence, 2000		0.142	0.073
HIV prevalence, 2010		0.100	0.053
ART prevalence, 2005		0.024	0.002
ART prevalence, 2007		0.039	0.004
ART prevalence, 2009		0.058	0.012
Total fertility rate, 2000		6.3	5.6
Total fertility rate, 2010		5.7	5.4

Notes: Data on CEB, CD, and TFR came from the 2010 DHS in each country [43, 44]. Estimates of HIV prevalence came from UNAIDS [45]. Data on number of women on ART and ART coverage come from WHO/UNICEF/UNAIDS [46] and national reports [47, 48]. Data on population (for the denominator in ART prevalence calculations) come from World Population Prospects [22]. We estimated past ART coverage by assuming a constant proportional increase from no coverage in 2004 to the levels reported by UNAIDS in 2009–2012

We estimated past ART coverage by assuming a constant proportional increase from no coverage in 2004 to the levels reported by UNAIDS in 2009–2012. We generated a point prediction and prediction interval for U5M for each country-age group-year observation, using standard statistical techniques [49]. We also compared our adjustments to adjustments generated by the predictive model in Ward and Zaba [16]. Because Ward & Zaba used a stable population model, it is not clear which year’s HIV prevalence is most appropriate for prediction. We used that of 10 years prior to the survey. This will likely overestimate the adjustment for women over 40 years old, but it should be reasonable for women aged 25–39 years.

## Results

Across the simulated populations, the mean HIV prevalence among women aged 15–49 across populations was 7% in 1990, 13% in 2000, and 9% in 2010 (this includes 107 populations without HIV) (Table 2). The highest HIV prevalence in any simulation was 40% in 2000. The mean ART coverage (the percent of women with a CD4 count under 200 cells/mm^3^ who are on ART) across simulations was 0% in 2004 and 42% in 2010. Mean ART prevalence (the proportion of all women aged 15–49 who are on ART) was < 0.1% in 2005 and 0.6% in 2009. The highest ART prevalence in any simulation was 4.4% in 2009. The mean TFR across simulations was 4.91 in 2000 and 4.30 in 2010. The HIV/AIDS death rate followed HIV prevalence with a lag of about 5 years.

**Table 2:** Outcomes for simulated populations, summary statistics

Variable	Mean	Std dev	Median	Min	Max
HIV prevalence, 1990	0.07	0.07	0.08	0	0.22
HIV prevalence, 2000	0.13	0.13	0.08	0	0.40
HIV prevalence, 2010	0.09	0.09	0.07	0	0.24
ART coverage, 2004	0	0	0	0	0
ART coverage, 2008	0.15	0.12	0.12	0	0.39
ART coverage, 2010	0.42	0.26	0.42	0	0.79
ART prevalence, 2005	0.0006	0.0011	0.0001	0	0.0057
ART prevalence, 2007	0.0029	0.0048	0.0006	0	0.0249
ART prevalence, 2009	0.0055	0.0088	0.0014	0	0.0440
Total fertility rate, 2000	4.91	1.67	4.64	2.86	6.89
Total fertility rate, 2010	4.30	1.65	4.16	2.44	6.18

Notes: Based on 4480 simulated populations. ART coverage is defined as the percent of women with a CD4 count under 200 who are on ART. ART prevalence is defined as the percent of women aged 15–49 who are on ART

For each of the 4480 simulated populations, we generated fourteen estimates of U5M, seven using surviving women (one estimate for each five-year age group from 15 to 19 to 45–49), and seven using surviving women and women who died from HIV/AIDS. Using those two sets of U5M estimates, we calculated 31,360 (7 * 4480) estimates of bias based on the difference between the unadjusted estimate (using reports from surviving women only) and the adjusted estimate (using reports from surviving women plus women who died from HIV/AIDS).

Table 3 shows the bias in indirect estimates across age groups; negative numbers indicate that unadjusted estimates were lower than adjusted estimates. The mean absolute bias was largest for estimates from women aged 35–39 and 40–44 (− 0.017) and smallest for estimates from women aged 15–19 and 20–24 (− 0.001). The largest absolute bias recorded was − 0.069 for estimates from women 35–39, meaning that the estimated U5M was 69 deaths per 1000 live births lower when using only reports from surviving women compared to reports from surviving women and women who died from HIV/AIDS.

**Table 3:** Bias in indirect estimates in 4480 simulated populations

Outcome variable	statistic	15–19	20–24	25–29	30–34	35–39	40–44	45–49
Absolute bias	*mean*	−0.001	− 0.001	− 0.005	−0.011	− 0.017	−0.017	− 0.013
	*std dev*	0.001	0.001	0.005	0.012	0.017	0.015	0.011
	*median*	−0.000	−0.001	−0.002	− 0.004	−0.013	− 0.016	−0.011
	*Min*	−0.009	−0.006	− 0.023	−0.049	− 0.069	−0.058	− 0.042
	*max*	0.002	0.001	0	0	0	0	0
Relative bias	*mean*	−0.6%	−1.5%	−4.4%	−7.7%	−9.7%	−8.8%	−5.6%
	*std dev*	1.0%	1.9%	5.6%	9.4%	10.2%	8.5%	5.5%
	*median*	−0.1%	−0.8%	−2.5%	−4.4%	−6.3%	−6.2%	− 4.4%
	*Min*	−5.0%	−9.5%	−23.7%	−36.8%	−40.5%	−31.6%	−22.6%
	*max*	0.9%	0.6%	0.0%	0.0%	0.0%	0.0%	0.0%
Yrs before survey that estimates pertain to	*mean*	1.2	2.6	4.1	6.0	8.0	10.4	13.7
Surviving women	*mean*	71,427	61,184	49,658	37,532	27,968	22,047	18,238
Women who died from HIV/AIDS	*mean*	72,539	62,697	53,827	45,099	37,243	30,994	25,212
Children ever born, surv women	*mean*	0.27	1.16	2.37	3.63	4.62	5.14	5.40
Children ever born, surv women + HIV deaths	*mean*	0.26	1.15	2.30	3.41	4.20	4.64	5.03
Dead children, surviving women	*mean*	0.09	0.10	0.12	0.14	0.15	0.18	0.22
Dead children, surv women + HIV deaths	*mean*	0.09	0.10	0.12	0.15	0.17	0.19	0.23
Ratio of HIV deaths to surviving women	*mean*	0.02	0.03	0.09	0.27	0.51	0.59	0.51

The mean relative bias was highest for estimates from women aged 35–39 (−9.7%), followed by estimates from women 40–44 (−8.8%) and women 30–34 (−7.7%). Mean relative bias was also substantial for estimates from 45 to 49 year olds (−5.6%) and 25–29 year olds (−4.4%). For the two youngest age groups the mean relative bias was −1.5% [20–24] and − 0.6% [15–19]. The largest recorded relative biases were − 40.5% for estimates from 35 to 39 year olds, −36.8% for estimates from 30 to 34 year olds and − 31.6% for estimates from 40 to 44 year olds, which appeared in simulated populations with the highest HIV incidence curves, yielding HIV prevalence of up to 40% in 2000. These populations also had relatively low U5M (120–130 deaths per 1000 live births).

The mean of the ratio of HIV deaths to the number of surviving women was highest for those aged 40–44 (0.59) followed by 45–49 and 35–39 (0.51), 30–34 (0.27), 25–29 (0.09), 20–24 (0.03) and 15–19 year olds (0.02). Comparing surviving women to surviving women and HIV deaths, the mean number of children ever born begins to diverge at age 25–29, and the mean number of dead children begins to diverge at age 30–34. On average, women who died from HIV had fewer births and more dead children.

**Fig. 3:**
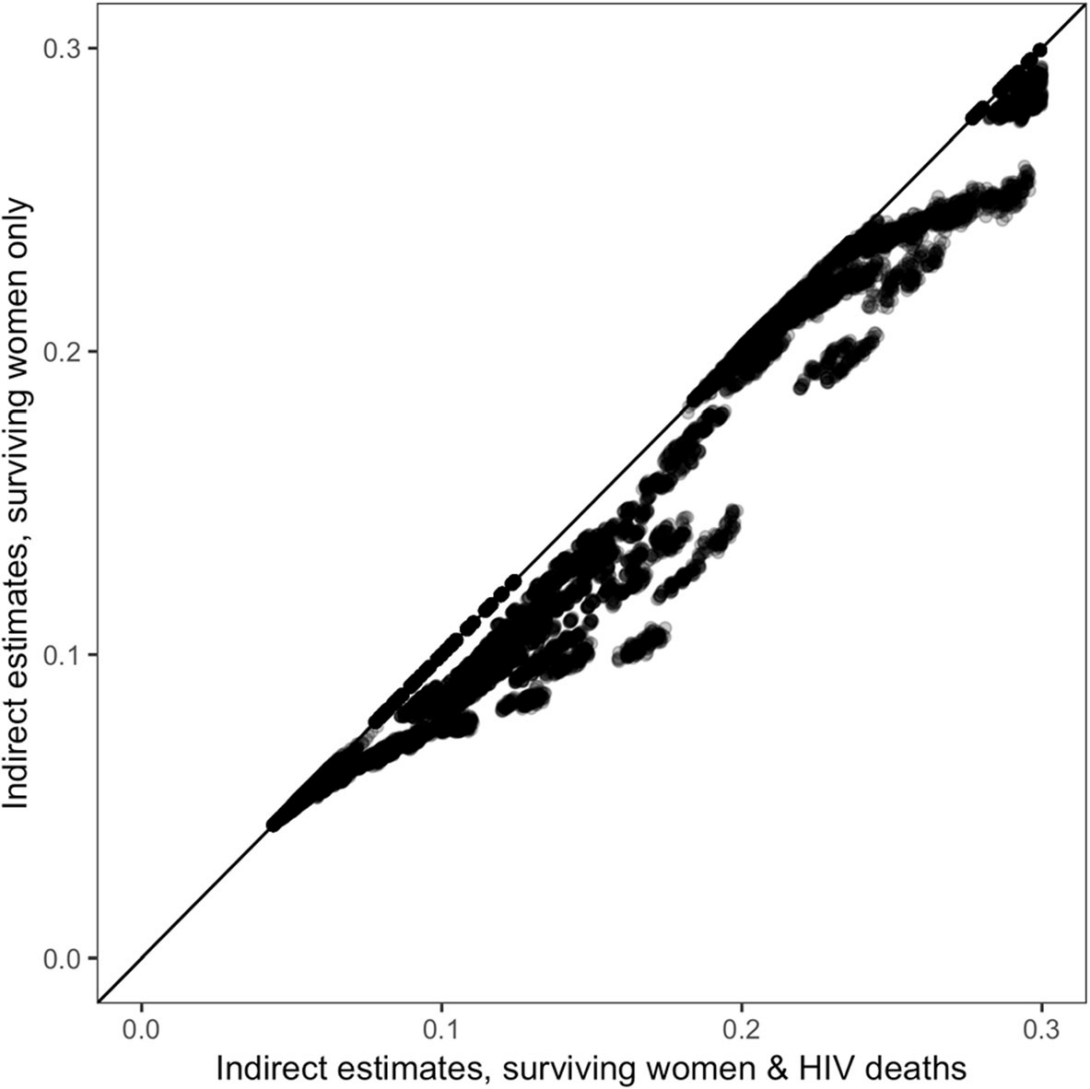
Indirect estimates from reports of surviving women versus indirect estimates from reports of surviving women and women who died from HIV/AIDS, based on 4480 simulated populations

Fig. 3 shows unadjusted and HIV-adjusted estimates across all simulated observations. Each point represents one age group-population specific estimate of U5M. There are 31,360 age group-population observations (one estimate per age group for 4480 simulated populations). Including the reports of women who died from HIV/AIDS increased the estimated 5q0 in all populations with HIV prevalence greater than zero.

Table 4 compares the prediction errors across the 13 models, both in-sample (using the entire dataset), and out-of-sample, as described above. No single model dominated across all error metrics. Focusing on the out-of-sample metrics, the generalized linear regression with alpha equal to 1 (i.e. lasso) had the lowest root mean square error, mean relative error, and median relative error. The generalized linear regression with alpha equal to 0.5 had lower root median square error. We used the lasso regression as our predictive model because it performed the best on the most metrics.

**Table 4:** Prediction errors from models to correct bias in indirect estimates of U5M

Method	Sample	Root mean square error	Root median square error	Mean relative error	Median relative error
Full Linear Model	in-sample	0.002973	0.001111	2.340	0.390
	out-of-sample	0.014922	0.004183	1.970	1.082
Forward Sel. BIC	in-sample	0.002988	0.001143	2.325	0.392
	out-of-sample	0.014914	0.004152	1.966	1.087
Forward Sel. AIC	in-sample	0.002975	0.001132	2.324	0.389
	out-of-sample	0.014925	0.004184	1.968	1.084
Backward Sel. BIC	in-sample	0.002974	0.001110	2.331	0.389
	out-of-sample	0.014923	0.004182	1.966	1.082
Backward Sel. AIC	in-sample	0.002974	0.001110	2.331	0.389
	out-of-sample	0.014923	0.004182	1.966	1.082
glmnet, alpha = 0	in-sample	0.003241	0.001135	2.571	0.406
	out-of-sample	0.003256	0.001150	1.100	0.382
glmnet, alpha = 0.5	in-sample	0.002985	0.001139	2.265	0.389
	out-of-sample	0.002968	0.001177	0.965	0.376
glmnet, alpha = 1	in-sample	0.002985	0.001137	2.314	0.391
	out-of-sample	0.002967	0.001175	0.960	0.374
PCR, ncomp = 20	in-sample	0.003989	0.002094	4.295	0.647
	out-of-sample	0.014700	0.005351	3.004	1.455
PCR, ncomp = 30	in-sample	0.003122	0.001430	1.789	0.407
	out-of-sample	0.014953	0.004504	1.868	1.101
PCR, ncomp = 35	in-sample	0.002994	0.001163	1.722	0.369
	out-of-sample	0.014925	0.004281	1.836	1.087
PLS, ncomp = 16	in-sample	0.002988	0.001154	1.844	0.382
	out-of-sample	0.014925	0.004264	1.872	1.087
PLS, ncomp = 32	in-sample	0.002973	0.001110	2.356	0.389
	out-of-sample	0.014922	0.004183	1.976	1.082

Note: *BIC* Bayesian Information Criterion, *AIC* Akaike Information Criterion, *glmnet* generalized linear model via penalized maximum likelihood, where alpha is the elastic-net penalty term, *PCR* principle components regression; *PLS* partial least squares, *ncomp* number of components

**Fig. 4:**
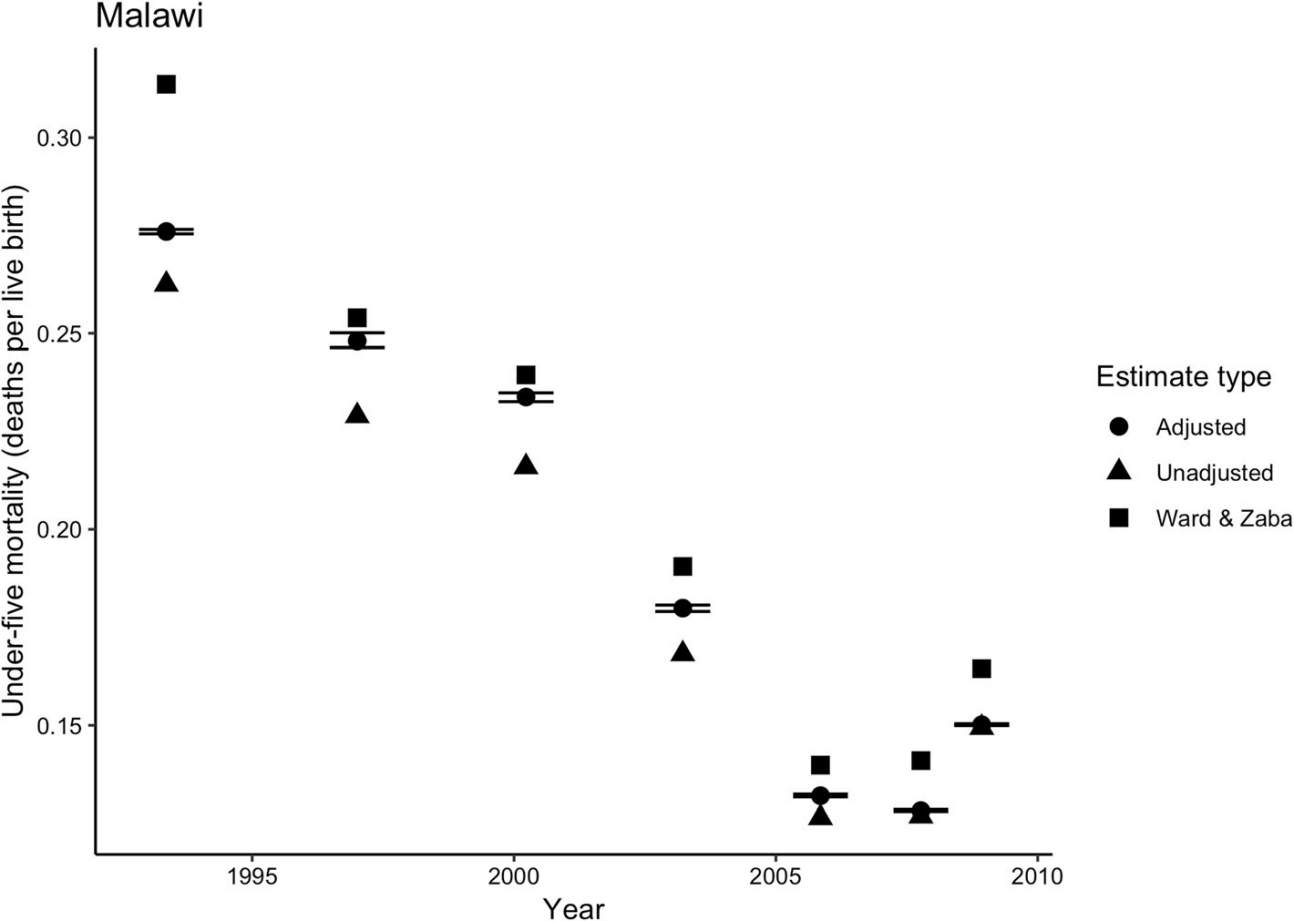
Under-five mortality estimates using CEB/CS from 2010 DHS for Malawi: unadjusted (crude), adjusted (corrected) using best-performing model, and using Ward & Zaba [16] model. Notes: Only the adjusted estimates include uncertainty intervals

**Fig. 5:**
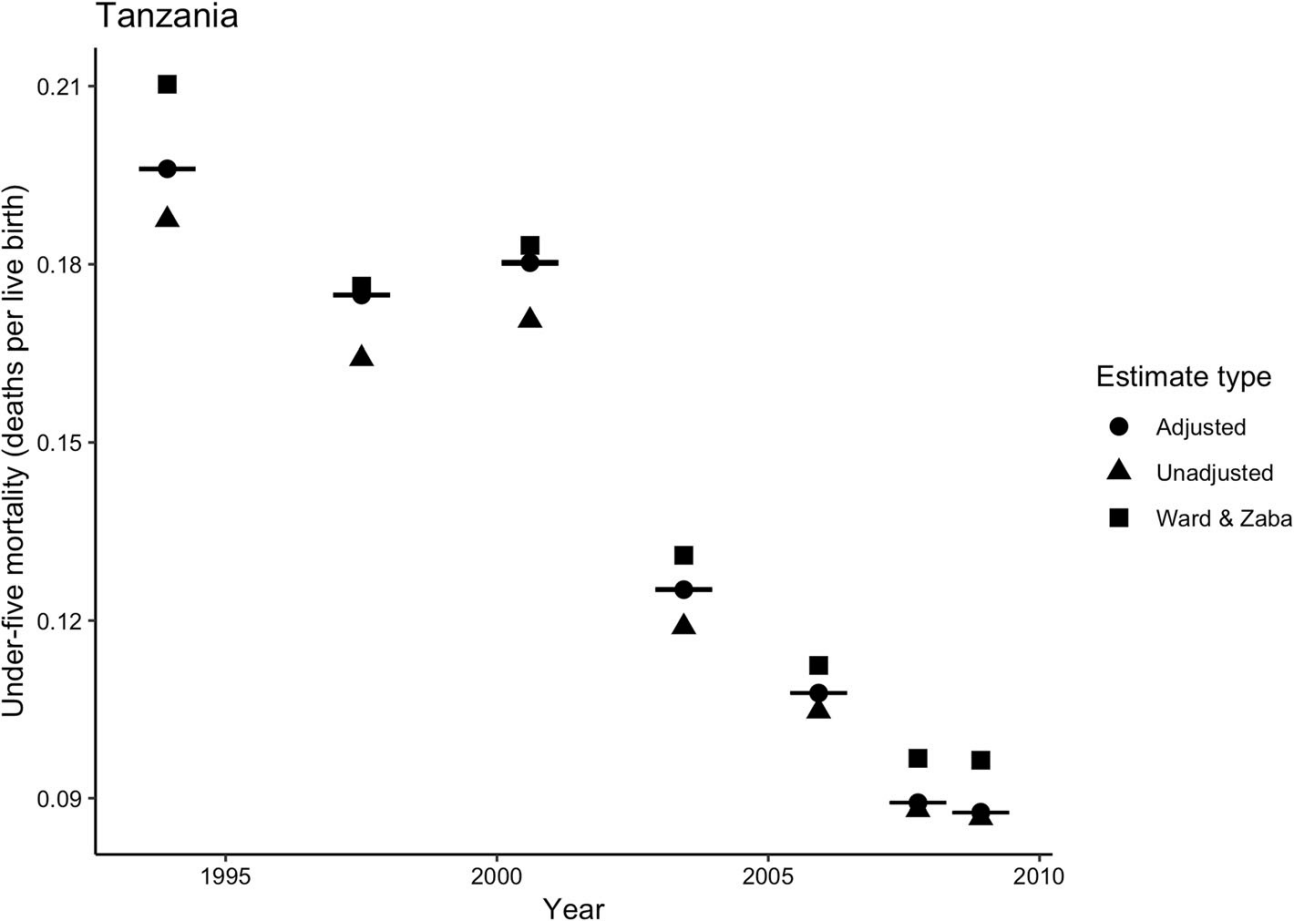
Under-five mortality estimates using CEB/CS from 2010 DHS for Tanzania: unadjusted (crude), adjusted (corrected) using best-performing model, and using Ward & Zaba [16] model. Notes: Only the adjusted estimates include uncertainty intervals

To assess whether the predictive model provides reasonable adjustments, we applied it to empirical data from 2010 in Malawi and Tanzania on CEB and CS, and estimates of HIV prevalence and ART prevalence. Figures 4 and 5 show the adjusted and unadjusted estimates of U5M for each country, along with adjustments from the Ward and Zaba [16] model. Note that the scale of the vertical axis changes across countries. For both countries, there were negligible differences between our adjusted estimates and the unadjusted estimates from the two youngest age groups (i.e. the two time points closest to the survey date, 2010). The relative adjustments for these age groups were 0.5–1.37%. Going further back in time, the adjusted and unadjusted estimates diverged among estimates from 25 to 29 year olds (2.9–4.2%, pertaining to 2006) and showed particularly large differences among estimates from 35 to 39 year olds (5.4–7.7%, 2001/2002) and from 40 to 44 year olds (6.1–7.7%, 1998/1999), while the difference between adjusted and unadjusted estimates from 45 to 49 year olds (4.4–4.9%, 1995/1996) were smaller. The largest absolute adjustment from our model was 0.0191 (19.1 deaths per 1000 live births), for estimates from 40 to 44 year olds in Malawi. The Ward and Zaba [16] adjustments were larger than our adjustments for all country-years.

## Discussion

Selection bias occurs in indirect estimates of U5M based on CEB and CS when the survival of children born to mothers who are not included in the survey differs from the survival of children whose mothers are included. In populations with high rates of HIV/AIDS, this selection bias can be significant, because a relatively large proportion of mothers die during their reproductive ages and their children die more frequently than other children due to the vertical transmission of HIV and the adverse effects of not having a living mother.

In this paper we presented an individual-based discrete time simulation model to measure and correct the bias in indirect estimates of U5M due to HIV/AIDS. The simulated populations were based on data and estimates from sub-Saharan Africa. We estimated bias by comparing indirect estimates from simulated reports of surviving women to estimates from simulated reports of surviving women and women who died from HIV/AIDS. We calculated bias in 4480 simulated populations, covering a range of peak HIV prevalence (0–40%), time between epidemic initiation and survey (25–35 years), ART coverage (0–79%), background U5M (50–290 deaths per 1000 live births), and TFR (2.4–6.9).

Our results showed negligible bias in estimates from 15 to 19 and 20–24 year olds. Unfortunately, this finding is of little practical value, since estimates based on reports of women at these ages are biased upwards for other reasons [50]. However, reports from surviving women aged 25 and older underestimated U5M by over two percentage points (over 20 deaths per 1000 live births), or, in relative terms, 24%. Bias was greatest in reports from 30 to 34, 35–39 and 40–44 year olds, reaching 69 deaths per 1000 births, a relative bias of 41%. The magnitude of the bias calculated by our model is somewhat difficult to compare to that found by Ward and Zaba [16] because of their use of a stable population model. They estimated that relative bias increased from − 1.2% to − 44.3% as the adult prevalence of HIV increased from 2.5 to 45%. That is generally consistent with the results of the present study, in which adult prevalence of HIV ranged from 0 to 40% and the relative bias ranged from 0% to − 41%. Also consistent with our results, Ward and Zaba found that estimates from women aged over 30 were more biased than estimates from women under 25. We found, however, that bias in estimates from women aged 45–49 was lower than in estimates from those aged 30–44. This was due to two related factors. First, as Ward and Zaba noted, stable population models assume that the level of age-specific incidence risks is constant over time. For any given level of prevalence, a stable population model will overestimate the exposure of older cohorts, because no actual population has been subject to constant incidence for such a long period. Second, HIV incidence in our simulated populations peaked between 1988 and 1998, 12 to 22 years before the simulated surveys. Women who were 45–49 in 2010 would have given birth to many of their children prior to peak HIV incidence.

Our analysis has several advantages over previous work. Unlike the only other study of bias in indirect estimates [16], we did not use a stable population model, but allowed HIV, mortality and fertility rates to follow the trajectories of selected countries, and we also included ART. Thus we used a larger variety of inputs and more recent empirical data than Ward and Zaba [16] and Hallet et al. [17]. In our simulations, the range of HIV prevalence was similar to that of Ward and Zaba, who used peak prevalence from 0 to 45%. We modeled background adult mortality using estimated _45_q_15_ from country-time periods corresponding to life expectancies from 47 to 64 years; Ward and Zaba allowed adult mortality to vary from a life expectancy of 41 to 67 years. It is difficult to compare our fertility rates to their fertility model as they reported only the range they used for the location (− 0.5 to 0.5) and spread (0.8 to 1.2) parameters of the relation system based on the Gompertz transformation of the Brass-Booth standard.

Our model also has several limitations. First, although the range of population characteristics was wider than in previous studies, the trajectories of HIV incidence, ART coverage, mortality rates and fertility rates considered here were a small fraction of all possible trajectories. The results of the predictive model should be applied with caution to population trajectories outside of the bounds explored in this study. Second, empirical data on the inputs required by the predictive model may not be available for some populations. In those cases, estimated inputs can be used. We encourage users to generate a range of bias estimates using a range of plausible estimated inputs (i.e. sensitivity analysis). Third, as in all models, our simulation included a number of simplifying assumptions, such as: use of a 1 year time step rather than continuous time; independence between the probability of giving birth and the probability of contracting HIV in a given time-step (although the probability of giving birth changes in time-steps following infection); use of only one set of age-specific HIV incidence ratios; independence of the probability of giving birth and CD4 count (although the former is influenced by HIV and ART status); independence of the effect of HIV infection on fertility and the duration of infection (this relationship is difficult to quantify [51]); independence of child survival and maternal survival, other than through vertical transmission of HIV; use of a single model life table to convert _n_q_0_ into _5_q_0_, which does not incorporate the effect of HIV on the age pattern of mortality [15, 52]; all vertical transmission occurs at birth; absence of variation in the effectiveness of ART in preventing vertical transmission; no drop-out once ART is initiated; and all women on ART take up PMTCT (and no women not on ART take up PMTCT). In most of these cases, we adopted these simplifying assumptions because they were expected to have relatively minimal effect on the main quantity of interest in this study, which was the HIV-related bias in indirect U5M rates; moreover, independent measurements of mortality, fertility and HIV rates showed that those rates were within acceptable ranges for our simulated populations (Table 2). Third, our study did not assess bias in indirect estimates due to factors other than HIV/AIDS. It is well-established that indirect methods applied to reports from women aged 15–19 (and in some cases women aged 20–24) tend to overestimate U5M, due to the higher risk of first births and the correlation between lower socioeconomic status and younger childbearing (Hill 1991).

HIV can also cause bias in direct estimation of U5M. Walker, Hill, and Zhao [18] found relative biases ranging from 1.1 to 26.5% across six African countries and time periods ranging from 1 to 5 to 11–15 years before the survey. They found that the largest biases were in estimates from 6 to 10 years before the survey (corresponding to indirect estimates from 30 to 44 year olds), and that biases in estimates from 11 to 15 years before the survey (corresponding to indirect estimates from 45 to 49 year olds) were slightly lower, which is consistent with the results that we found. Hallett et al. [17], applying direct methods to prospective cohort data from rural Zimbabwe, measured a relative underestimate of 9.8% in U5M for the period 0–7 years before the survey, a period during which HIV prevalence fell from 23 to 18% among the study population, with minimal ART coverage, in a population with relatively low U5M (0.0671). Taking as inputs 18% HIV prevalence in the year of the survey, 20.5% 10 years earlier, 23% 20 years earlier, with a baseline U5M of 0.0671, our model predicts a relative underestimate of 15.4% for 4 years prior to the survey (estimates from 25 to 29 year olds). This is reasonably close to the Hallett et al. given the probable overestimate of prevalence used for 20 years prior to the survey, and the sensitivity of relative bias measures at low levels of U5M.

## Conclusion

In populations affected by HIV/AIDS, indirect estimates of U5M can be significantly biased. Our predictive model allows scholars and practitioners to correct that bias using commonly measured population characteristics. Policies and programs based on indirect estimates of U5M in populations with generalized HIV epidemics may need to be reevaluated after accounting for bias in indirect estimates.

## Data Availability

The data and code are freely available at https://github.com/jquattro/hiv-childmort-bias. A user-friendly web application to correct indirect estimates is available at johnquattrochi.com/bias.

## Abbreviations

AIDS: Acquired immune deficiency syndrome
ART: Anti-retroviral therapy
ASFR: Age-specific fertility rates
CD: Children dead
CD4: Cluster of differentiation 4
CEB: Children ever born
DHS: Demographic and health survey
HIV: Human immunodeficiency virus
IE: Indirect estimates
IGME: UN Inter-agency Group for Child Mortality Estimation
MDG: Millennium Development Goals
MMR: Maternal mortality ratio
PMTCT: Prevention of mother-to-child transmission
SDG: Sustainable Development Goals
TFR: Total fertility rate
U5M: Under-5 mortality
UNIAIDS: Joint United Nations Programme on HIV/AIDS
WDI: World Development Indicators
